# Peptide Composition of Stroke Causing Emboli Correlate with Serum Markers of Atherosclerosis and Inflammation

**DOI:** 10.3389/fneur.2017.00427

**Published:** 2017-09-01

**Authors:** Neal M. Rao, Joseph Capri, Whitaker Cohn, Maram Abdaljaleel, Lucas Restrepo, Jeffrey A. Gornbein, William H. Yong, David S. Liebeskind, Julian P. Whitelegge

**Affiliations:** ^1^David Geffen School of Medicine, University of California, Los Angeles, Los Angeles, CA, United States; ^2^University of California, Los Angeles, Los Angeles, CA, United States

**Keywords:** proteomics, stroke, stroke etiology, mass spectrometry, mechanical thrombectomy, thrombus proteomics

## Abstract

**Introduction:**

The specific protein composition of stroke-causing emboli is unknown. Because ischemic stroke has a varied etiology, it is possible that the composition of the thrombus from which an embolus originated will have distinctive molecular characteristics reflective of the underlying pathophysiology. We used mass spectrometry to evaluate the protein composition of retrieved emboli from patients with differing stroke etiologies and correlated the protein levels to serum predictors of atherosclerosis.

**Methods:**

Emboli from 20 consecutive acute stroke patients were retrieved by thrombectomy during routine stroke care. Thrombus proteins were extracted, digested, and multidimensional fractionation of peptides was performed. Fractionated peptides underwent nano-liquid chromatography with tandem mass spectrometry. Spectra were searched using Mascot software in which results with *p* < 0.05 (95% confidence interval) were considered significant and indicating identity. The results were correlated to A_1_C, low-density lipoprotein (LDL), and erythrocyte sedimentation rate (ESR) taken on admission.

**Results:**

Eleven patients had atrial fibrillation, four had significant proximal vessel atherosclerosis, two were cryptogenic, and three had other identified stroke risk factors (left ventricular thrombus, dissection, endocarditis). Eighty-one common proteins (e.g., hemoglobin, fibrin, actin) were found in all 20 emboli. Serum LDL levels correlated with Septin-2 (*r*_s_ = 0.78, *p* = 0.028), Phosphoglycerate Kinase 1 (*r*_s_ = 0.75, *p* = 0.036), Integrin Alpha-M (*r*_s_ = 0.68, *p* = 0.033) and Glucose-6-phosphate dehydrogenase (*r*_s_ = 0.63, *p* = 0.05). Septin-7 levels inversely correlated to ESR (*r*_s_ = −0.84, *p* = 0.01). No significant protein correlations to A_1_C or tPA use were found.

**Conclusion:**

Our exploratory study presents mass spectrometry analysis of thrombi retrieved from acute stroke patients and correlates the thrombus proteome to clinical features of the patient. Notably, we found proteins associated with inflammation (e.g., Integrin Alpha-M) in emboli from patients with high LDL. Although these findings are tempered by a small sample size, we provide preliminary support for the feasibility of utilizing proteomic analysis of emboli to discover proteins that may be used as markers for stroke etiology.

## Background and Significance

Stroke is currently the fifth leading cause of death and the leading cause of disability in the US, with over 800,000 strokes per year. Approximately 10–40% of these patients, depending on work up, may be categorized as “cryptogenic”—with no determined cause (1). As the determination of etiology is essential to preventing further stroke, we must seek to improve diagnostic precision. The goal of our exploratory study is building the groundwork to develop a new method of determining stroke etiology through proteomic analysis.

**Figure 1:**
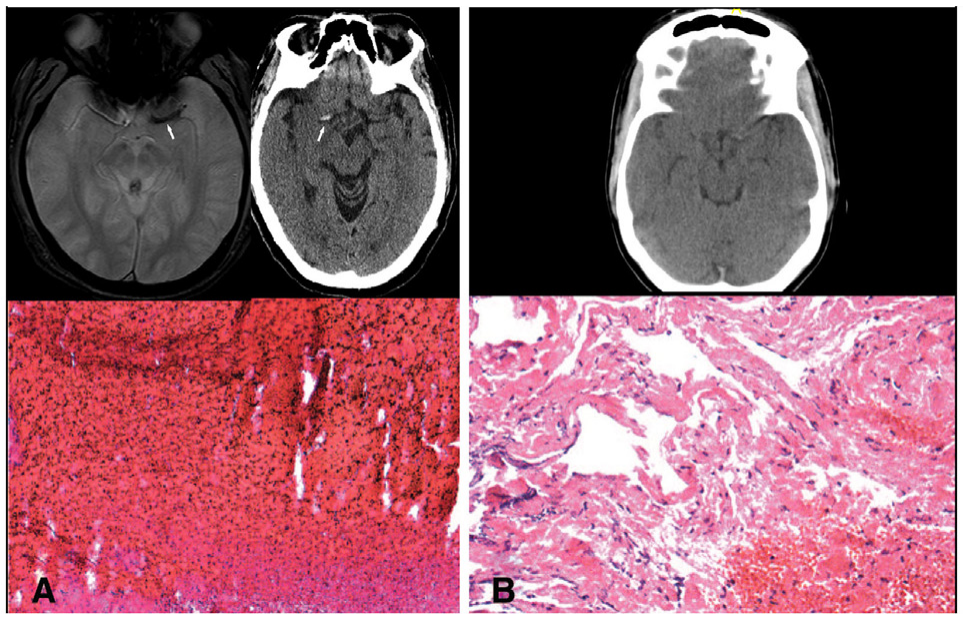
**(A)** Blooming artifact on gradient echo MRI sequence and hyperdense artery sign on CT with representative red blood cell rich thrombus. **(B)** Lack of significant hyperdense artery sign with representative fibrin rich thrombus.

With the advent of mechanical thrombectomy, only within the past decade have we been able to directly study a stroke-causing thrombus outside of an autopsy. Histopathologic studies of retrieved thrombi have correlated gross composition to the radiographic finding of hyperdense middle cerebral artery sign (Figure 1) (2). The density of this thrombus signal on CT has been correlated with revascularization success rate (3). These studies indicate that changes in even the gross histopathology of the thrombus can have important clinical implications. As we elucidate the exact contents of stroke-causing thrombi, the connection between etiology, thrombus composition, treatment, and outcome will become clearer. In this study, we tested the use of a protein extraction and mass spectrometry method to uncover the composition of these thrombi down to the peptide level. We then investigated correlations between these uncovered proteins and clinical characteristics of the patients. Ultimately, this technique will be used to uncover peptides from tissue of origin that may be trapped within the clot as it formed. Such peptide markers may be used as a diagnostic tool to determine the precise etiology of the stroke.

## Research Design and Methods

### Patient Selection

We analyzed thrombi from 20 consecutive patients who underwent mechanical thrombectomy for acute ischemic stroke. Patient charts were retrospectively reviewed and initial erythrocyte sedimentation rate (ESR), hemoglobin A_1_C (A_1_C) and low-density lipoprotein (LDL) values were recorded. These laboratory values were chosen as they were measured on admission for nearly all of the patients. Clinical characteristics were recorded as present if they were pre-existing on admission or uncovered through standard inpatient work up. Risk factors without judgment of causality are listed in Table 1. Clinical details and MRI or CT images taken on admission as part of routine care were evaluated independently of the histopathology and proteomic results to judge the most likely stroke etiology using the A-S-C-O-D phenotypic classification of stroke (4). Patients were designated as “cryptogenic” if no clear etiology of stroke was determined from inpatient work up, which at a minimum included cardiac monitoring, transthoracic echocardiogram, and imaging of the brain, cervical and cerebral blood vessels. Due to low sample size, the study was underpowered to detect differences based on etiologic distinction.

**Table 1:** Patient demographics.

Demographics	
Age, mean ± SD	67.85 ± 17.9
Female	13 (65%)
Received tPA	12 (60%)
Atrial fibrillation/cardiac abnormalities	11 (55%)
Proximal vessel atherosclerosis	4 (20%)
Diabetes	3 (15%)
Cryptogenic	2 (10%)
Other risk factors	3 (15%)
Low-density lipoprotein level, mean ± SD	89.3 ± 51.4
Erythrocyte sedimentation rate level, mean ± SD	26.9 ± 27.8
A_1_C, mean ± SD	5.8 ± 0.67

*Patients were designated as cryptogenic if no clear etiology of stroke was determined from inpatient work up, which at a minimum included cardiac monitoring, transthoracic echocardiogram, and imaging of the cervical and cerebral blood vessels. “Other risk factors” included left ventricular thrombus, dissection, and endocarditis*.

### Thrombus Preparation

The retrieved thrombus was rinsed in saline and split into two portions. One half was preserved in formaldehyde for histopathologic analysis as described in our prior histopathologic studies (2, 5). The other half was frozen at −80°C until analysis by mass spectrometry, as below.

On the day of analysis, the frozen samples were thawed and disrupted in lysis buffer using a mechanical homogenization technique (sonication). The proteins are then extracted using TRIzol standard protocol and undergo tryptic digestion. Multidimensional fractionation of the peptides is performed using reverse phase [C(18)] and strong cation exchange (SCX) StageTips. Each sample was run along with a pooled sample from all 20 patients in order to serve as an internal control.

### Mass Spectrometry Analysis

The fractionated peptides underwent nano-liquid chromatography with tandem mass spectrometry (nLC–MS/MS) analysis. nLC–MS/MS with Collision-induced dissociation (CID) was performed on an Orbitrap XL integrated with an Eksigent nano-LC. A prepacked reverse-phase column with a dimension of 75 µm × 20 cm containing resin (Biobasic C18, 5-µm particle size, 300-Å pore size) was used for peptide chromatography and subsequent CID analyses. ESI conditions using the nano-spray source for the Orbitrap were set as follows: capillary temperature of 220°C, tube lens 110 V, and a spray voltage of 2.3 kV. The flow rate for reverse-phase chromatography is 0.5 µl/min for loading and 400 nl/min for analytical separation (buffer A: 0.1% formic acid, 3% ACN; buffer B: 0.1% formic acid, 100% ACN). Peptides were resolved by the following gradient: 0–40% buffer B over 180 min and then returned to 0% buffer B for equilibration of 20 min. The Orbitrap was operated in data-dependent mode with a full precursor scan at high resolution (60,000 at *m/z* 400) and 10 MS/MS experiments at low resolution on the linear trap while the full scan was completed. For CID the intensity threshold was set to 5,000, where mass range was 350–2,000. Spectra were searched using Mascot software in which results with *p* < 0.05 (95% confidence interval) were considered significant and indicating identity. This protocol has been described in further detail in a prior publication by co-authors Capri and Whitelegge (6).

### Statistical Analysis

We computed Spearman correlations (*r*_s_) between each protein and the continuous ESR, A_1_C, or LDL and ranked the proteins by the absolute value of their correlations. The Spearman correlation was computed since the underlying relationship was monotone but not linear. To control for false positives from multiple testing, we used the Hochberg criteria to assess significance. Due to the small sample size for most proteins, multivariable analyses were not feasible. Also for this reason, proteins were compared to ESR, A_1_C, and LDL as values for these variables were present in nearly every patient.

### Ethical Aspects of the Research

The UCLA Institutional Review Board (IRB) has approved the abovementioned study. We have taken the following measures to ensure minimal risk to the patients and their private health information. The thrombus material was retrieved during routine clinical care for patients undergoing mechanical thrombectomy and would otherwise have been discarded. Research on this discarded material had no effect on or alteration to standard clinical care, and thus consent was not requested. Clinical data were entered into an encrypted, secure server in a locked room to minimize risk of exposing patient information. All samples were coded and stored without patient identifiers. The UCLA IRB determined this to be a minimal risk study.

## Results

The patients ranged from 4 to 85 years old with a median of 69.5. One thrombus was retrieved from a pediatric patient who suffered a stroke at age 4. The next youngest patient was 52. 13 (65%) were female, 12 (60%) received tPA, 11 (55%) had atrial fibrillation, 4 (20%) had significant proximal vessel atherosclerosis, 2 (10%) were cryptogenic, and 3 had other identified significant stroke risk factors (left ventricular thrombus, dissection, endocarditis). Patient demographics are listed in Table 1. Eighty-one common proteins (e.g., hemoglobin, fibrin, actin) were found in all 20 emboli. Top proteins correlating to A_1_C, LDL, and ESR are shown in Tables 2–4. Serum LDL levels correlated with Septin-2 (*r*_s_ = 0.78, *p* = 0.028), Phosphoglycerate Kinase 1 (*r*_s_ = 0.75, *p* = 0.036), Integrin Alpha-M (*r*_s_ = 0.68, *p* = 0.033) and Glucose-6-phosphate dehydrogenase (*r*_s_ = 0.63, *p* = 0.05) (Table 2). Septin-7 levels inversely correlated to ESR (*r*_s_ = −0.84, *p* = 0.01). Band 3 Anion Transport Protein levels correlated to ESR (*r*_s_ = 0.56, *p* = 0.03) (Table 3). No significant protein correlations to A_1_C (Table 4) or tPA administration were found.

**Table 2:** Peptides correlating to patient low-density lipoprotein levels.

Protein name	#Patients present	Correlation (*r*_s_)	*p*-Value
Septin-2	10	0.780	0.0277
Phosphoglycerate kinase 1	10	0.754	0.0356
Integrin alpha-M	12	0.684	0.0333
P31946_2 (unidentified peptide)	10	0.652	0.0781
Glucose-6-phosphate 1-dehydrogenase	12	0.636	0.0505
Dolichyl-diphosphooligosaccharide-protein glycosyltransferase	10	0.632	0.0893
Mitochondrial superoxide dismutase	13	0.603	0.0517

**Table 3:** Peptides correlating to patient erythrocyte sedimentation rate levels.

Protein name	#Patients present	Correlation (*r*_s_)	*p*-Value
Septin-7	10	−0.839	0.0146
Band 3 anion transport protein	17	0.561	0.0326
Apolipoprotein A-I	15	−0.555	0.0513
Annexin A3; Annexin	13	−0.564	0.0710

**Table 4:** Peptides correlating to patient A_1_C levels.

Protein name	#Patients present	Correlation (*r*_s_)	*p*-Value
Haptoglobin	11	0.620	0.0744
Cathepsin G	11	0.604	0.0832
Myotrophin	11	0.563	0.1092
Glia maturation factor gamma	11	0.554	0.1157
Annexin A3; Annexin	11	0.540	0.1258

A full list of proteins and peptides found in these studies can be requested by e-mailing the corresponding author, Neal M. Rao, MD.

## Discussion

In our study, several of the proteins correlating with LDL values were enzymes involved in metabolism (PGK1, G6PD, and DDOST)—an association that makes physiologic sense. Interestingly, two of the proteins that correlated to high levels of LDL, integrin alpha-M and mitochondrial superoxide dismutase (Mn-SOD), have been associated with inflammation. This is congruent with proteomic analysis of atherosclerotic plaques that have also found elevated levels of Mn-SOD in unstable plaques (7). It is also released from mitochondria during apoptosis. These results support the feasibility of mass spectrometry to detect differences in thrombus peptide composition.

Proteomic analysis of stroke-causing thrombi may be an important diagnostic tool to determine the underlying cause of stroke in each individual patient. Due to the age and comorbid medical risk factors of our stroke patient population, such as diabetes, heart disease, renal dysfunction, and atherosclerosis; currently, it is often impossible to definitively determine which of the contending etiologies ultimately resulted in the stroke. For instance, in the situation of stroke patients with atrial fibrillation, they are generally of the age where internal carotid artery stenosis is found as well. In these cases, it may be difficult to ascertain whether the source of the thrombus was arteroembolic from an unstable carotid, or cardioembolic from a thrombus formed by atrial fibrillation in the heart. The treatment to reduce stroke risk in the setting of carotid stenosis may entail surgery or stenting followed by an antiplatelet agent, while the treatment for atrial fibrillation would be anticoagulation. However, if we were able to determine that the thrombus came from the heart and that the carotid disease is an asymptomatic red herring, we may be able to spare the patient surgery and the additional hemorrhage risk of antiplatelet therapy on top of anticoagulation with warfarin.

Proteomic analysis of carotid plaque material retrieved during carotid endarterectomy has revealed a secretome—a mix of proteins secreted by unstable plaque (7, 8). If a thrombus is formed on such a plaque, in theory these proteins may be incorporated into this thrombus and, thus, may be detected. Likewise, a thrombus formed in the atria may incorporate proteins unique to atrial membranes. Thus, with the available mass spectrometry technology, we could have a test to differentiate between arterioembolic and cardioembolic thrombi in our mechanical thrombectomy patients. In this pilot study, we demonstrate the feasibility of proteomic analysis by mass spectrometry as a method to differentiate thrombi from patients with varying clinical characteristics. Future studies, with larger sample size will be needed to establish this technique as a valid diagnostic procedure.

### Study Limitations

The results of this study are tempered by small sample size, which may result in false positives among the several thousand peptides analyzed. Due to low sample size, the study was underpowered to detect differences based on A-S-C-O-D phenotypic classification. No significant protein correlations to A_1_C or tPA were found—this may also have been due to low sample size. One thrombus was from a pediatric stroke patient with likely cardioembolic stroke from congenital cardiac abnormalities. As we may gain important information from studying thrombi from diverse patient populations, we included analysis of this clot in the study. However, we acknowledge that this thrombus may have considerable differences from thrombi from standard populations. In addition, we suspect that only the core of the clot will likely have formed on the tissue of origin, and the remainder likely forming *in situ* around this nucleus after it has lodged in a vessel. In subsequent studies, we will sample protein variation across the clot to determine the initial nidus, as this may be most enriched with peptides from the tissue of origin. Furthermore, we are currently analyzing emboli from atherosclerotic etiology compared to those from atrial fibrillation as this analysis may uncover diagnostic peptide markers for differentiating the two conditions.

## Conclusion

We present a unique mass spectrometry analysis of emboli retrieved from acute stroke patients and correlate the thrombus proteome to clinical features. Notably, we found proteins associated with inflammation (Integrin Alpha-M, Mn-SOD) in emboli from patients with high LDL. Although these findings are tempered by a small sample size, we provide preliminary support for the feasibility of utilizing proteomic analysis of emboli to discover proteins that may be used as markers for stroke etiology.

## Ethics Statement

This study was carried out in accordance with the recommendations of the UCLA IRB (OFC OF HUMAN RESEARCH PROTECTION PROGRAM, IRB #12-00184). The study was determined as exempt from written consent as all data were from retrospective chart review, without any direct patient contact. All thrombus material was collected as part of routine clinical care and would otherwise be discarded material.

## Author Contributions

NR: primary, corresponding author. Project inception, design, and analysis of data. JC and WC: operation of mass spectrometry and data analysis. MA: histopathologic analysis of clot material. LR: data review and project mentorship. JG: statistical analysis. DL: data review and project mentorship. WY: oversight of histopathologic analysis of clot material. JW: data review, project mentorship, and mass spectrometry analysis.

## Conflict of Interest Statement

The authors declare that the research was conducted in the absence of any commercial or financial relationships that could be construed as a potential conflict of interest.
